# Correction: da Costa et al. Establishment of a Temperature-Sensitive Model of Oncogene-Induced Senescence in Angiosarcoma Cells. *Cancers* 2020, *12*, 395

**DOI:** 10.3390/cancers13092015

**Published:** 2021-04-22

**Authors:** Adilson da Costa, Michael Y. Bonner, Shikha Rao, Linda Gilbert, Maiko Sasaki, Justin Elsey, Jamie MacKelfresh, Jack L. Arbiser

**Affiliations:** 1 Department of Dermatology, Emory University School of Medicine, Atlanta, GA 30322, USA; adilson_Costa@hotmail.com (A.d.C.); srao30@emory.edu (S.R.); lcgilbert@yahoo.com (L.G.); Justin.elsey@emory.edu (J.E.); jpbower@emory.edu (J.M.); 2 Department of Post-Graduate Studies, Instituto de Assistência Médica ao Servidor Público Estadual, Sao Paulo 04029-000, SP, Brazil; 3 Department of Medical Biochemistry & Biophysics, Karolinska Institutet, 17177 Solna, Sweden; Michael.Bonner@ki.se; 4 Atlanta Veterans Affairs Medical Center, Decatur, GA 30033, USA; mpapke@emory.edu; 5 Department of Pathology, Emory University School of Medicine, Atlanta, GA 30322, USA; 6 Winship Cancer Institute of Emory University, Atlanta, GA 30322, USA

The authors wish to make the following corrections to this paper [1]:

The following words in bold face are to be added: In Section 2.1 “Cells”, lines 3 and 4: 1% complex of antibiotics/L-glutamine (**stock =** 10,000 IU/mL penicillin, 10,000 μg/mL streptomycin, and 29.2 mg/mL **L-glutamine**; Mediatech Inc., Manassas, VA, USA); in Section 2.4 “Western Blot (WB)”, lines 5 and 7: “**tri**-buffered saline” should be changed to “**Tris**-buffered saline” on both lines.

In the original article, there was a mistake in Figure 3, where the MW of β-actin was incorrectly recorded as 65 kd, when it should be changed to 45 kd. The new Figure 3 is listed below: 
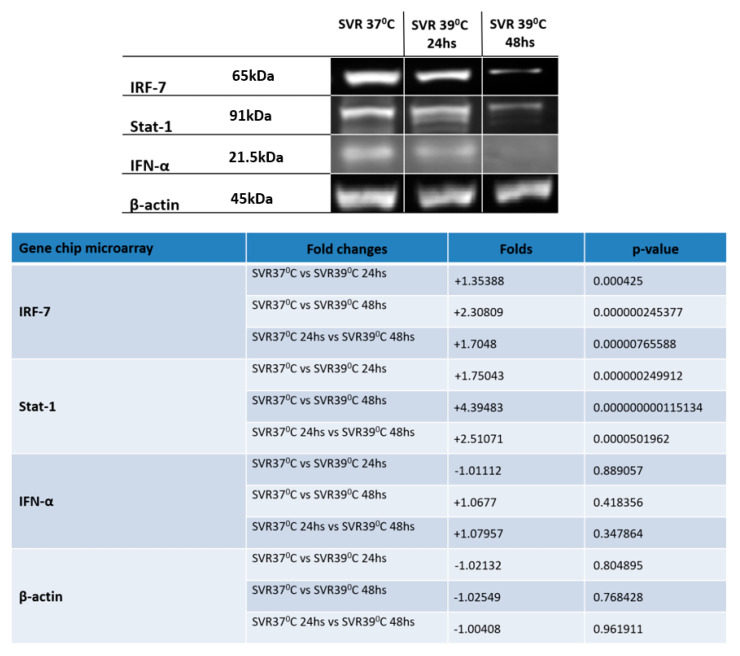


In Figure 4, ‘**B-actin**’ should be changed to ‘**β-actin**’. The new Figure 4 is listed below: 
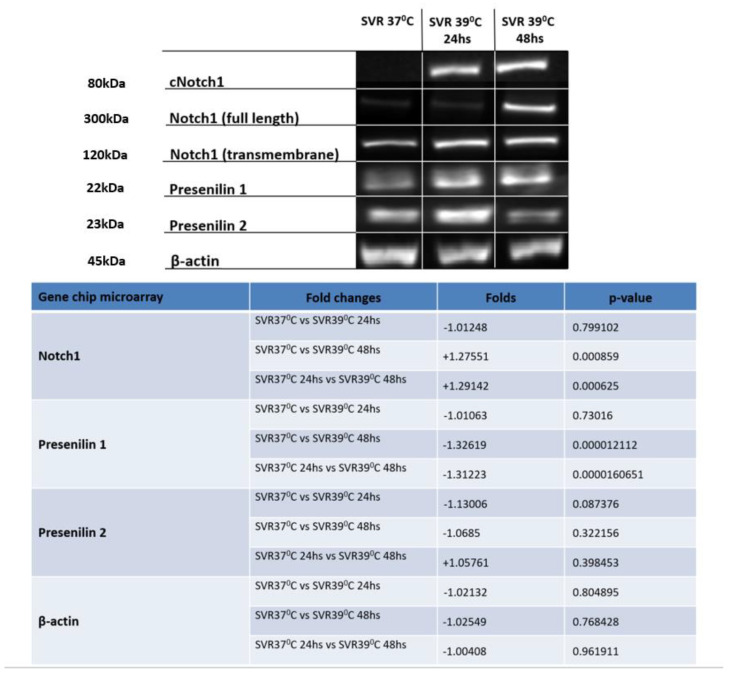


In Figure 5, the SDHA band was accidently duplicated and listed as p53. The duplicated band has been removed. The new Figure 5 is listed below: 
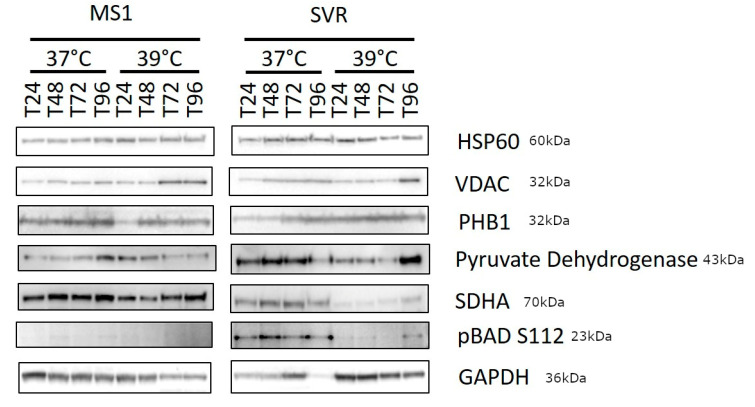


The p53 figure represents a duplication of the SDHA Western blot, and thus should be deleted. Since we are not using the p53 data, we request the deletion of the following sentences: “**p53 is dephosphorylated in SVR cells upon shifting to the nonpermissive temperature in ras transformed SVR cells, but not in MS1 cells, suggesting that this is not a nonspecific heat shock event, but that it is induced by oncogenic ras**” in the last paragraph of Section 3 “Result” and the sentence “**p53 is rapidly phosphorylated in SVR cells upon shift to 39 °C (Figure 5) and it is well known that phosphorylated p53 translocates to the mitochondria and may mediate apoptosis** [46]” in Section 4 “Discussion and Conclusions” should be removed. Reference [47] and [48] should thus be re-numbered to [46] and [47], respectively.

The authors apologize for any inconvenience caused and state that the scientific conclusions are unaffected. The original article has been updated.
